# Supplementary material for: Capturing ultrafast molecular motions and lattice dynamics in spin crossover film using femtosecond diffraction methods
Source: Nat Commun. 2025 Feb 27;16:2043. doi: 10.1038/s41467-025-57202-0 (PMC11868369; doi:10.1038/s41467-025-57202-0)
Supplement: Supplementary file 1 — Supplementary Information [file 41467_2025_57202_MOESM1_ESM.pdf]

Supplementary Information

Capturing ultrafast molecular motions and lattice dynamics in spin crossover film using femtosecond diffraction methods

Vinci *et al*.

Contents

Supple. Note 1. Debye-Waller effect correction 2
Supple. Note 2. Fluence dependence measurement. 3
Supple. Note 3. Fit of the UED and XRD data. 4
Supple. Note 4. Time-dependent model of atomic motions. 5
Supple. Note 5. Lattice temperature estimation. 11
Supple. Note 6. Excitation fraction estimations. 13
Supple. Discussion 1. Analysis of observed oscillations in the UED and XRD results 17

Supple. Note 1. Debye-Waller effect correction

The photoinduced difference diffraction map has a close match with the thermally-induced difference map, which shows the phase transition from the low-spin (LS) to the high-spin (HS) state, in Fig. 2b, c. However, it is worth noting that at +2 ps in the photoinduced intensity changes of Bragg peaks (Fig. 2c), many Bragg peaks show a decrease in intensity rather than an increase. This might suggest the presence of a Debye-Waller (DW) effect on an ultrafast timescale in the ultrafast electron diffraction (UED) data, rather than ultrafast molecular switching. The DW effect is characterized by a decrease in Bragg peak intensity due to thermal atomic motions, which are related to the random mean square displacement of atoms from their average positions. This effect has been observed in numerous UED studies and is typically induced by the pump laser.

To investigate the role of DW effect in the UED data, we calculate it using the photo-Wilson plot[1,2]. This plot represents the natural logarithm of the quasi-steady-state intensity of 20 selected Bragg peaks against their d-spacing squared (Supple. Eq. 1).

$$\ln\left[\frac{I(t)}{I_0}\right] = -\frac{4}{3}\pi^2\left(\frac{1}{d^2}\right)\langle\delta u^2(t)\rangle \qquad \text{Supple. Eq. (1)}$$

where $I(t)$ is the intensity of the Bragg peaks at a given time t after photoexcitation (in this case, $t = +2$ ps). I_0 is the Bragg peak intensity without excitation, d is the distance between crystal planes, and $\langle\delta u^2(t)\rangle$ is the mean-square atomic displacement.

In Supple. Fig. 1, we present the fitting results of the photo-Wilson plot of UED at +2 ps after photoexcitation.

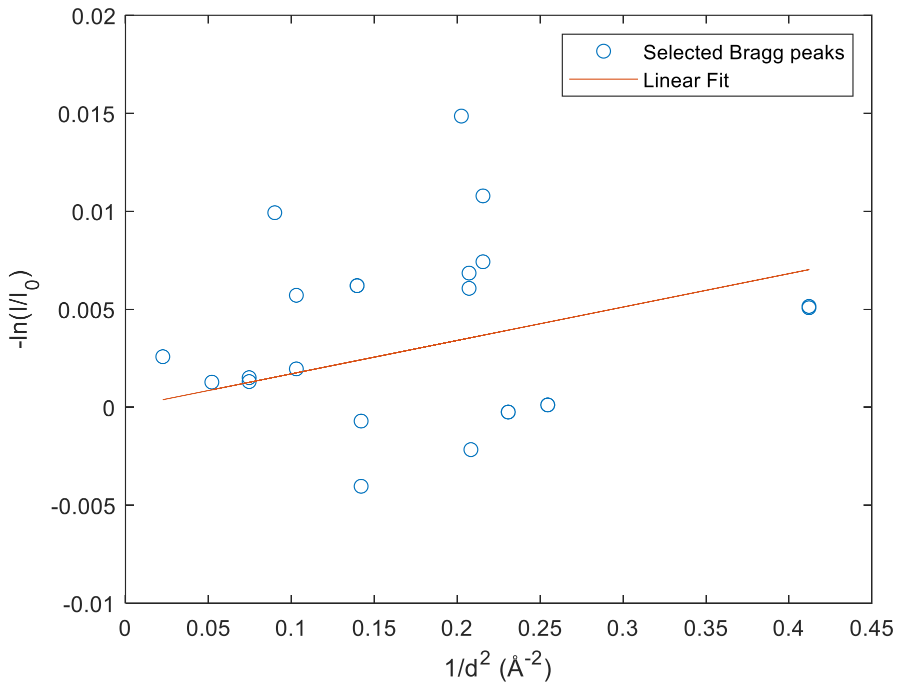

Supple. Fig. 1. Debye-Waller effect calculation for UED data at +2 ps. Natural logarithm of the quasi-steady-state intensity ratio *vs.* (1/d)-spacing squared. The red line is a linear fit. Source data are provided as a Source Data file.

Supple. Note 2. Fluence dependence measurement. A fluence dependence measurement was performed during the UED experiment. In Supple. Fig. 2, we plot the mean absolute relative intensity change (MARIC) in Bragg peaks as a function of pump laser fluence. Linear dependence does not extrapolate to the (0,0) point due to signal-to-noise level of the MARIC. The fluences we used in both measurements are in the linear range of the fluence dependence measurement (Supple. Fig. 2) and are low enough to avoid complications through multiphoton absorption processes. The fluence used is also consistent with the previous studies on SCO solids[3–5]. In both UED and X-ray diffraction (XRD) experiments, we used the fluence below the sample damage threshold. A higher damage threshold observed in XRD can be likely attributed to the lower repetition rate in XRD (10 Hz) as compared to that in UED (100 Hz).

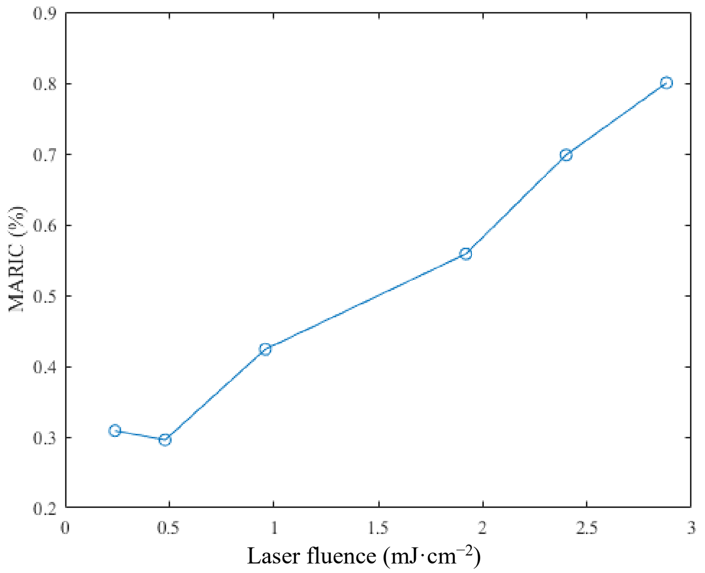

Supple. Fig. 2. Mean absolute relative intensity change (MARIC) of Bragg peaks at +2 ps as a function of laser fluence. Source data are provided as a Source Data file.

Supple. Note 3. Fit of the UED and XRD data. In this work, the fitting was used to investigate time traces of UED and XRD data. For changes in intensity of Bragg peaks shown in Fig. 2d and 3b, they were fitted with two time constants, since two relaxation processes of intersystem crossing (ISC) and intramolecular vibrational relaxation (IVR) were suggested by the distinct behaviors of some Bragg peaks in these two processes and previous literature:

$$f(t) = \frac{1}{2}\sum_{n} a_n \left(1 + \mathrm{erf}\left(\frac{t}{\sigma\sqrt{2}}\right) \exp\left(-\frac{t}{\tau_n}\right) \exp\left(\frac{\left(\sigma/\tau_n\right)^2}{2}\right)\left[1 + \mathrm{erf}\left(\frac{t - \sigma^2/\tau_n}{\sigma\sqrt{2}}\right)\right]\right) \quad \text{Supple. Eq. (2)}$$

where σ is the instrumental response, and τ_n (n = 1, 2) are the time constants.

In Fig. 5a, the radial position shifts of 200 and 020 Bragg peaks were fitted with monoexponential function using Supple. Eq. 2 with only one time constant.
In Fig. 5a and 5b, oscillations observed in changes in intensity and radial shift of peak position of Bragg peaks were fitted using a global fitting model:

$$f(t) = a \exp\left(-\frac{t}{\tau}\right) \cos\left(\frac{2\pi}{T}(t - \varphi)\right) \quad \text{Supple. Eq. (3)}$$

where τ is the damping time and T is the period of the oscillations.

Supple. Note 4. Time-dependent model of atomic motions.

Both crystal structures at low temperature (LT, T = 300 K) and high temperature (HT, T = 373 K) are in *Pbca* space group. The molecule structure of **1** in the LS state is presented in Supple. Fig. 3.

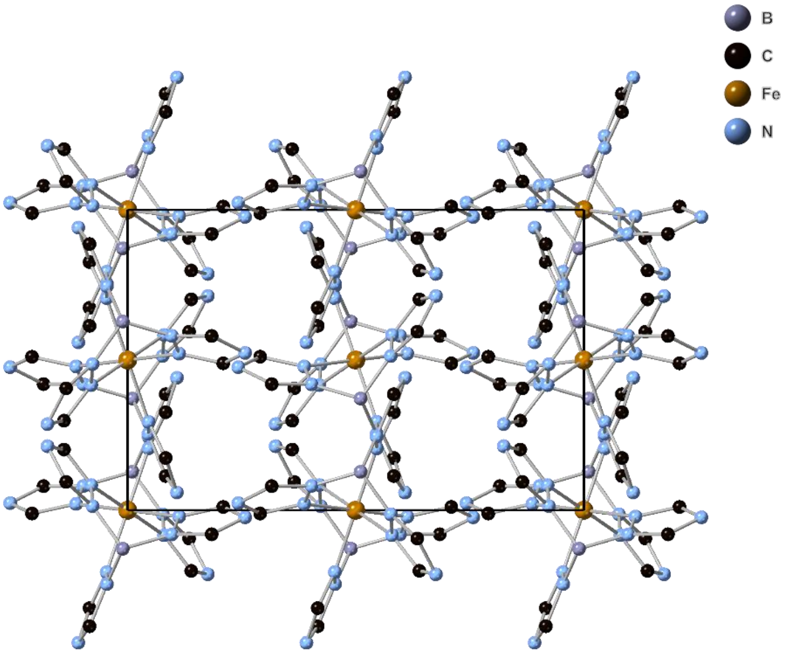

Supple. Fig. 3. **Crystal structure of 1 viewed along the *X* crystallographic direction.** Black spheres are carbon atoms; blue nitrogen atoms; brown iron(II) atoms; purple boron atoms.

For our structural modeling, we focused on the time-resolved intensity changes of 80 Bragg peaks from the *hk0* family observed in UED results, complemented by 32 Bragg peaks from the *hkl* family (where l = 1 or 2) in XRD results. The a, b, and c unit-cell parameters were derived from the time-resolved radial positions of these Bragg peaks in the XRD data. The excitation fraction was determined by laser fluence and photoinduced structural changes (Supple. Note 6). We selected time delays from −1 to +2 ps, before the onset of volume expansion, which affects Bragg peak intensities. A singular value decomposition (SVD) filter using the first five components was applied to these changes prior to fitting the parameterized model[6,7].

Our approach utilizes a parameterized molecular model to interpret the structural dynamics of molecular crystals based on UED and XRD data. This methodology has been used in studying ultrafast structural dynamics with UED data containing Bragg peaks from only one crystal plane in various systems, including two iron(II) spin crossover systems[3,5], but it is to our best knowledge the first time to use this methodology with combined UED and XRD data. Supple. Fig. 4 outlines the workflow, which involves identifying potential key modes from the literature and chemistry knowledge, generating a grid of proposed excited state structures by linear displacement along these key modes, and calculating the correlation between the experimental

Bragg peak intensity changes and those simulated from the proposed excited state structures. The Pearson correlation coefficient equations in previous publications[6,7]:

$$\mathrm{PC}_{\mathrm{sim,exp}}(p,t) = \frac{\mathrm{cov}\left(\eta_{\mathrm{sim}}(\mathbf{p},\mathbf{q}_j),\eta_{\mathrm{exp}}(t,\mathbf{q}_j)\right)}{\sigma_{\mathrm{sim}}\sigma_{\mathrm{exp}}} \qquad \text{Supple. Eq. (4)}$$

$$\eta_{\mathrm{sim}}(\mathbf{p},\mathbf{q}_j) = \frac{F_{\mathrm{sim}}(\mathbf{p},\mathbf{q}_j)}{F_{\mathrm{off}}(\mathbf{q}_j)} - 1 \qquad \text{Supple. Eq. (5)}$$

$$\eta_{\mathrm{sim}}(\mathbf{p},\mathbf{q}_j) = \frac{\sqrt{I_{\mathrm{sim}}(t,\mathbf{q}_j)}}{\sqrt{I_{\mathrm{off}}(\mathbf{q}_j)}} - 1 \qquad \text{Supple. Eq. (6)}$$

$$\mathrm{cov}\left(\eta_{\mathrm{sim}}(\mathbf{p},\mathbf{q}_j),\eta_{\mathrm{exp}}(t,\mathbf{q}_j)\right) = \frac{1}{M}\sum_{j=1}^{M}\left(\eta_{\mathrm{sim}}(\mathbf{q}_j)-\bar{\eta}_{\mathrm{sim}}(\mathbf{q}_j)\right)\left(\eta_{\mathrm{exp}}(\mathbf{q}_j)-\bar{\eta}_{\mathrm{exp}}(\mathbf{q}_j)\right) \qquad \text{Supple. Eq. (7)}$$

$$\sigma_{\mathrm{sim}} = \sqrt{\frac{1}{M}\sum_{j=1}^{M}\left(\eta_{\mathrm{sim}}(\mathbf{q}_j)-\bar{\eta}_{\mathrm{sim}}(\mathbf{q}_j)\right)^2} \qquad \text{Supple. Eq. (8)}$$

$$\sigma_{\mathrm{exp}} = \sqrt{\frac{1}{M}\sum_{j=1}^{M}\left(\eta_{\mathrm{exp}}(\mathbf{q}_j)-\bar{\eta}_{\mathrm{exp}}(\mathbf{q}_j)\right)^2} \qquad \text{Supple. Eq. (9)}$$

PC is Pearson correlation coefficient; **p** is a vector of parameter values to atomic coordinates; η is response ratio (relative intensity change for a given reflection); t is time delay point; **q** is reciprocal lattice vector; j are the indices of the sampled Bragg peaks; F is the structure factor; cov is the covariance; M is the total number of Bragg peaks used in the analysis. Since small fraction of the molecules undergo structural changes, the simulated changes in structure factor can be written as:

$$F_{\mathrm{sim}} = nF_{\mathrm{sim,exc}} + (1-n)F_{\mathrm{sim,off}} \qquad \text{Supple. Eq. (10)}$$

n is excitation fraction obtained in Supple. Note 6; $\mathrm{F}_{\mathrm{sim,exc}}$ is the structure factor of proposed simulated structure; $\mathrm{F}_{\mathrm{sim,off}}$ is the structure factor of initial ground state structure.

During the modeling process, we made the following assumptions to ensure accuracy and coherence in our analysis:

1. Constant excitation fraction: The excitation fraction was determined by laser fluence and photoinduced structural changes at +2 ps (Supple. Note 6). We assumed that the excitation fraction of HS molecules remains constant throughout the timescale of the modeling. This assumption is supported by previous optical studies on this sample[8] and on similar spin crossover (SCO) complexes[9].

2. Coherent and homogenous structural changes: We posited that the structural transition from LS to HS is coherent and homogeneous. This hypothesis is backed by several studies that observed similar behavior[10–12].
3. Consistent symmetry: Both LS in LT and HS in HT were assumed to remain in the orthorhombic *Pbca* space group, with no symmetry change during the modeling timescale. This is corroborated by time-resolved data showing no disappearance or appearance of Bragg peaks, supporting the consistency of the symmetry.
4. Thermally-induced HS state: We assumed that the structure of the photoinduced HS state is similar to the thermally-induced HS state at high temperature (373 K). This is based on findings from previous ultrafast electron diffraction (UED) studies[3,5] and consistent with results from X-ray scatter measurements[13].
5. Fixed iron(II) centers: The iron(II) centers were assumed to remain fixed in their positions as found in the low-temperature (LT) crystal structure.

These assumptions provided a robust framework for modeling the structural dynamics accurately, ensuring the reliability of our conclusions.

In this study, we identified symmetric Fe-ligand elongation and ligand rotation towards the high-spin (HS) state as the two primary structural dynamics. Our simplified model, incorporating these two parameters, provided the best fit for the UED and XRD data. The selection of these key structural dynamics is based on the study of the molecular structure at LS and HS states, theoretical calculations on low-frequency modes, and previous research on SCO systems. Our choice of the structural mode allowed us to keep the number of degrees of freedom to a minimum and capture the most important features of the photoinduced dynamics, while avoiding overfitting and chemically unreasonable structures.

1. One major consideration on the choice of structural model is based on the molecular structure and its changes during the SCO. As shown in Fig. 1a and 1b, the molecule exhibits high symmetry, justifying a reduction in degrees of freedom. In Figure 4a, we overlaps the LS and HS state structures, aligned at the iron center, highlighting the structural changes during the transition can be broken down to symmetric Fe-ligand elongation and ligand rotation on both $HB(tz)_3$ ligand.
2. Within the classic description of transition-state processes, each molecule would have a distinct many-body potential energy surface, with distinct modes reflecting the different degrees of freedom needed to describe the structural dynamics[10]. In practice, molecular structural dynamics are often dominated by a few low-frequency, large-amplitude modes, which describe the essential dynamics of the system.
3. Our theoretical calculations revealed vibrational modes between 70 cm^{-1} and 200 cm^{-1}, with results uploaded for reference (Supple. Data 1). Among these, the Fe−ligand symmetric breathing (Supple. Movie. 1) and bending modes (Supple. Movie. 2) correspond to the Fe-ligand elongation and rotation, contributing most to the structural changes. Thus, at least two vibrational modes define the reaction coordinate. Optical and X-ray absorption studies confirm these modes sufficiently describe the dominant SCO pathway.

4. The Fe−ligand expansion results directly from SCO due to weaker Fe bonding in the HS state[14], and it is assigned to the Fe−ligand breathing mode. We attribute the Fe−ligand rotation to the Fe−ligand bending mode, as this mode involves motions aligned with changes in the N−Fe−N angle of the $HB(tz)_3$ ligand, resulting in a less strained ligand structure. This bending mode plays a crucial role in relaxing the photoexcited molecule within the high-spin (HS) potential[11,15,16]. Given the ligand's rigidity, previous UED studies on SCO materials suggest that atomic motions around the ligand during SCO are strongly linked to changes in the electron distribution at the metal center[6,8]. Consequently, the ligand motion is treated as a single group.
5. Other low-frequency modes, such as ligand torsion (Supple. Movie. 3) and out of phase Fe−ligand stretching (Supple. Movie. 4), are reported to be irrelevant during vibrational cooling[11,15,16]. Adding these modes in the structural modelling could lead to overfitting rather than presenting important structural dynamics.

Based on the above considerations and assumptions, we parameterized the transition from the LS to the HS state. First, we converted the Cartesian coordinates of the HS state structure at HT into the LT unit cell to obtain a modified HS structure. We then decomposed the structural change into two components: the elongation of the Fe-ligand bonds and the rotation of the ligands. By linearly interpolating these atomic motions, we parameterized the transition from the LS to the HS state.

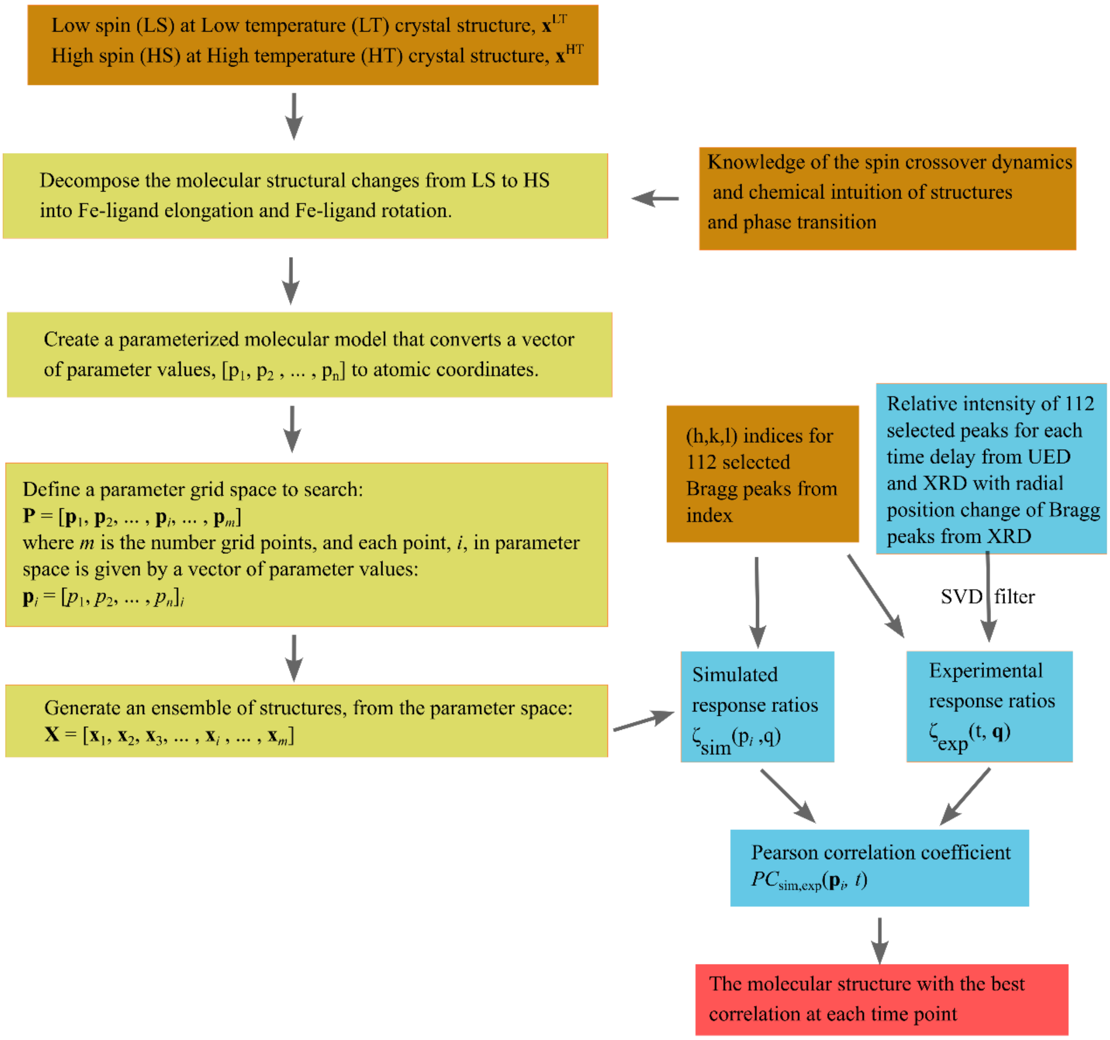

Supple. Fig. 4. Workflow of the time-dependent model of atomic motions.

The structure factors in the diffraction pattern reflect the atomic positions within the molecular structure. The Pearson correlation coefficient, ranging from −1 (indicating a perfect negative linear relationship) to +1 (indicating a perfect positive linear relationship), measures the similarity between the experimentally observed changes in Bragg peak intensities and those calculated by simulations of the proposed excited-state structures. Supple. Fig. 5 illustrates cross-section of the maximum Pearson correlation coefficient for two key structural modes: Fe−ligand elongation (Supple. Fig. 5a) and ligand rotation (Supple. Fig. 5b), at +2 ps (t = +2 ps). These results are based on Bragg peaks obtained from both UED and XRD (blue lines) or XRD alone (red lines).

The blue lines in Supple. Fig. 5 show the solution based on Bragg peaks obtained from UED and XRD results. They are well behaved since they clearly show only one maximum with high Pearson correlation coefficient, approximately matching the HS structure (Fig. 4b). In contrast, the red lines represent solution based on Bragg peaks obtained from XRD results alone, which show less consistent behavior. In Supple. Fig. 5a, multiple maxima with comparable Pearson correlation coefficient values are observed, all lower than those obtained from the combined UED and XRD solution, indicating reduced accuracy and uncertainty in structural refinement.

In Supple. Fig. 5b, the maximum peak for ligand rotation exhibits a lower Pearson correlation coefficient and a broader peak width, reflecting reduced similarity to the experimental data and larger associated errors. The FWHM of the peak is used to calculate an error bound in Fig 3c in the manuscript.

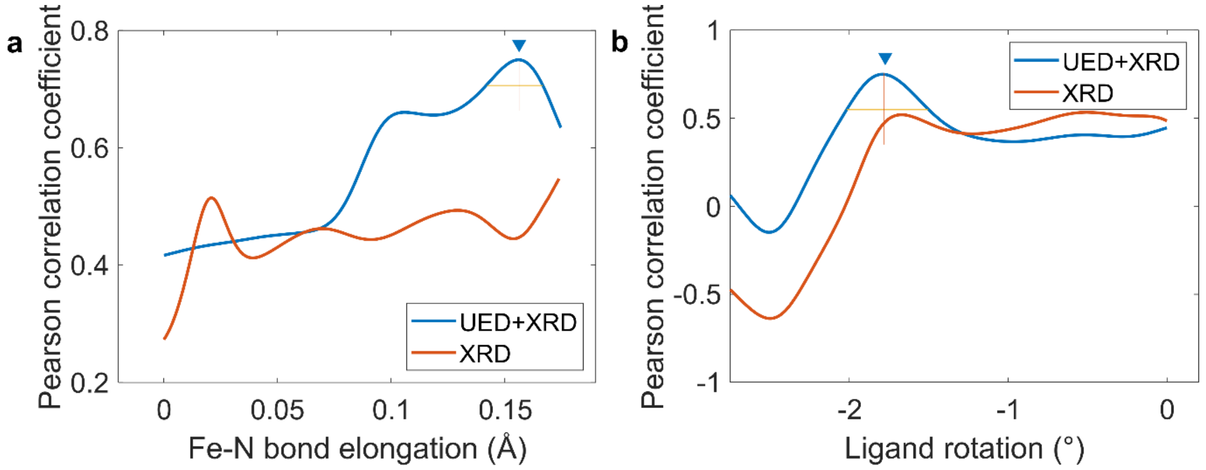

Supple. Fig. 5. **Cross-section of the maximum Pearson correlation coefficient of the two key structural modes at +2 ps. a**, Cross-section of the maximum Pearson correlation coefficient of Fe-ligand elongation. **b**, Cross-section of the maximum Pearson correlation coefficient of ligand rotation. The blue lines show the Pearson correlation coefficient based on both UED and XRD results. The red lines show the Pearson correlation coefficient based on XRD results only. a-b, the blue marks show the maximums of the Pearson correlation, and the yellow lines show prominence and FWHM of the peaks. Source data are provided as a Source Data file.

Supple. Fig. 6 provides further visualization of Bragg peak intensity changes simulated from the proposed excited-state structures with the best correlation at +2 ps, compared with the experimental changes shown in Fig. 2b and 2c. Supple. Fig. 6a displays the static diffraction pattern, with colored circles indicating the positions of the selected Bragg peaks from Fig. 2d. Supple. Figs. 6b and 6c present the experimental intensity changes from thermally induced SCO and photoinduced SCO at +2 ps, respectively. Supple. Fig. 6d shows the simulated Bragg peak intensity changes based on the proposed excited-state structures with the best correlation at +2 ps, as identified in Supple. Fig. 5. The simulated changes in Supple. Fig. 6d closely align with the transient changes observed at +2 ps following photoexcitation (Supple. Fig. 6c) and the thermally induced changes (Supple. Fig. 6b). This similarity further demonstrates the good fit between the simulated and the experimental data. While most Bragg peaks exhibit similar changes in both cases, some show subtle differences. We believe these deviations may be from the low excitation fraction and limited signal-to-noise ratio in the Supple. Fig. 6c to show changes in the diffraction pattern, but the kinetic traces (Fig. 2d) clearly show the similar behaviors between thermal equilibrium and the +2 ps transient changes. For example, the 020 Bragg peak (blue circle) displays an intensity increase in the thermally induced diffraction changes (Supple. Fig. 2b) but shows no clear change in the +2 ps transient diffraction changes (Supple. Fig. 2c). However, as shown in Fig. 2d, the 020 peak undergoes a rapid intensity increase followed by a gradual decline.

Therefore, in discussing the complementary nature of XRD and UED results for structural refinement at +2 ps, the extensive *q*-range and the large number of Bragg peaks from the *hk*0 family in UED enhance the spatial resolution of the model. This reduces the risk of underfitting or overfitting that could occur if relying solely on the limited Bragg peaks available in XRD. The combined data from UED and XRD provides a robust basis for modeling, enabling confident assignment of structural dynamics and lattice responses.

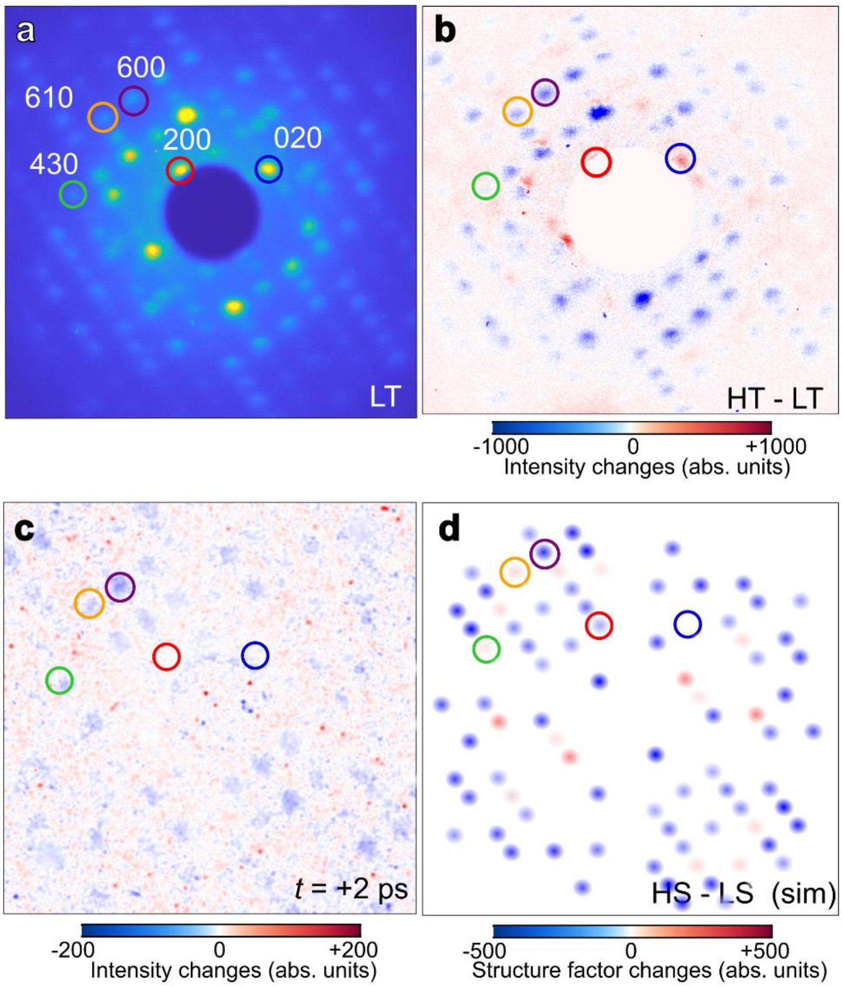

Supple. Fig. 6. **Experimental and simulated changes** in the diffraction pattern. **a**, Static electron diffraction pattern at 300 K low-temperature (LT). **b**, Difference between the diffraction patterns of LT and 373 K high-temperature (HT) phases. **c**, Photoinduced changes in the diffraction pattern measured at +2 ps after photoexcitation at 267 nm at 300 K. **d**, Difference between low spin (LS) structure determined from X-ray diffraction and excited state structure at +2 ps in the LT unit cell.

Supple. Note 5. Lattice temperature estimation. In the time-resolved XRD experiment, we observed clear peak shift of the 200 and 020 Bragg peaks at strain waves process (tens of picosecond), showing the thermal expansion of the *a*- and *b*- unti-cell parameters. To estimate the maximum temperature increase of the lattice (ΔT_{latt}) we adopt a reported method[9]:

$$\Delta T_{\mathrm{latt}} = \frac{F V_{\mathrm{cell}} N_{\mathrm{A}}}{Z C_{\mathrm{P}}^{\mathrm{mol}} t} (1 - e^{-\alpha t}) \qquad \text{Supple. Eq. (11)}$$

where F is the incident excitation fluence, V_{cell} is the average volume of the unit cell, N_{A} is the Avogadro constant, Z is number of molecule per unit cell, C_{P}^{mol} is the molar heat capacity[17], α is the absorption coefficient of the material and t is its thickness. The estimated temperature increase yields a value of approximately 60 K, resulting from the laser power in the XRD measurements. Note that this estimate assumes full energy conversion to heat and does not consider radiative losses, therefore it is likely an upper limit.

Furthermore, we extracted a linear dilation in the thin film along the X and Y crystallographic directions due to ordinary thermal expansion[18] by assuming similar behaviors of thermal expansion in out-of-equilibrium and equilibrium states. The changes due to molecular switching are removed based on the estimated excitation fraction. Given the peak shifts of 200 and 020 Bragg peaks at +100 ps and linear dilatation coefficients of a unit-cell parameters ($4.61 \cdot 10^{-4}$ Å$\cdot$K^{-1}) and b unit-cell parameters ($3.21 \cdot 10^{-4}$ Å$\cdot$K^{-1}), the estimated temperature increases are approximately 47 and 35 K, respectively. The consistency between these two estimated temperatures increases and the above estimation supports our observation that on the 100-picosecond timescale, the dominant effect behind the lattice-volume changes is the laser heating.

Supple. Note 6. Excitation fraction estimations. The excitation fraction (fractional population of photoexcited HS molecules) (ΔX_{HS}) is critical for the data analysis. Two independent methods were applied to estimate ΔX_{HS} value. We first estimated ΔX_{HS} using the laser excitation conditions (knowing the optical absorption properties of **1**) using the following formula:

$$\Delta X_{HS} = \frac{FV_{cell}}{ZE_{ph}t}(1 - e^{-\alpha t}) \qquad \text{Supple. Eq. (12)}$$

where F is the laser fluence, V_{cell} is the unit-cell volume, Z is the number of molecules per unit cell, E_{ph} is the photon energy, t is the film thickness, and α is the absorption coefficient of the SCO material at the excitation wavelength. This method assumes that each absorbed photon excites one LS molecule into HS (quantum efficiency of unity)[19]. Note also that from optical spectroscopic measurements[8], the excitation fraction in the films of **1**, is known to be constant during the first 3 ns after photoexcitation.

On the other hand, ΔX_{HS} can be also determined by a complementary way. This method is possible since both thermally-induced and fully-relaxed photoinduced HS states have a similar structure, thus their structure factors almost coincide: $F_{exc}(t_\infty, \mathbf{q}) \approx F_{HT}(\mathbf{q})$ and then:

$$\Delta X_{HS} = \frac{F_{exc}(t_\infty, \mathbf{q}) - F_{LT}(\mathbf{q})}{F_{HT}(\mathbf{q}) - F_{LT}(\mathbf{q})} \qquad \text{Supple. Eq. (13)}$$

where $\mathbf{q}$ is reciprocal-lattice vector, F_{HT} is the structure factor of Bragg peaks in the HS phase, and F_{LT} is the structure factor of Bragg peaks in the LS phase. A distribution of ΔX_{HS} from different Bragg peaks is expected since this estimation is sensitive to the signal-to-noise ratio and Bragg conditions of diffraction peaks. We performed this calculation for Bragg peaks from both XRD and UED results, resulting in a well-defined normal distribution, with the median of this distribution used as the representative value for ΔX_{HS}. Supple. Fig. 7 shows the normal distribution of the UED data at +2 ps as an example.

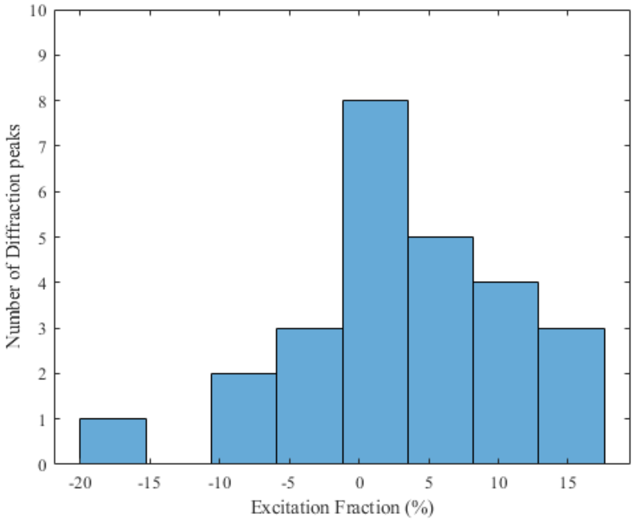

Supple. Fig. 7. Histogram of the values of the fraction of photoexcited molecules ΔX_{HS} determined from UED data.

For the thermal differences in UED results, the temperature changes from 300 to 373 K, so the thermal differences obtained in the UED experiment present a near 100% HS fraction. For the

UED experiment, ΔX_{HS} estimated from the incident laser fluence is 2.8 %. For ΔX_{HS} estimated from the structure factor, from the experimental data and literature, we consider that the photoexcited molecules have reached HS state after 1.5 ps. Therefore, we take t_∞ = 1.5 ps < t = 2 ps, before strain waves start to dominate the dynamics. The ΔX_{HS} estimated by this method is 3.2 %, which is consistent with the value estimated from the excitation conditions.

In the temperature-dependent XRD study, LT and HT curves were measured at 313 and 353 K, respectively, which is close to the transition temperature of the film (T_C = 336 K). This rather small temperature difference helps to minimize the contributions from the regular thermal expansion and maximize the contributions from the molecular switching from LS to HS state. From the previous optical characterization of this sample, a change of the fraction of HS molecules by 85 % is expected between 313 and 353 K[20]. For the XRD experiment, ΔX_{HS} estimated from the incident laser fluence is 5.7 %. The value of ΔX_{HS} on the ultrafast timescale (1.5 ps < t < 2 ps) obtained from the structure factors is 6.8 %, in reasonable accordance with the above independent determination.

In Supple. Fig. 8, we show the kinetic traces of the 200 and 020 Bragg peaks in the low-q range from −1 to +4 ps, obtained from both UED and XRD measurements, although the keV XFEL and MeV electron sources result in significantly different Ewald sphere radii for diffraction. The kinetic traces of the 200 and 020 peaks exhibit remarkably similar dynamic features, confirming that both UED and XRD measurements capture the same structural dynamics. The roughly twofold difference in the relative changes in peak intensity between the UED and XRD measurements in Supple. Fig. 8 aligns with the ratio of their excitation fractions, indicating that the excitation conditions are within the linear single-photon regime, as described in Supple. Note 2.

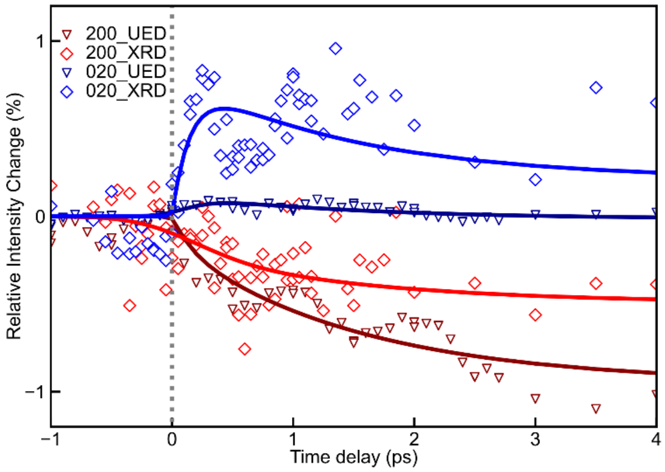

Supple. Fig. 8. **Kinetic traces of 200 and 020 Bragg peaks from −1 to +4 ps from both UED and XRD measurements.** The solid lines show the results of the global fit to a two-exponential decay functions (Supple. Equation 2). Source data are provided as a Source Data file.

For ΔX_{HS} in ns timescale, we use the changes in intensity at +37 ns (t_∞ = 37 ns) for the estimation. Using this method, the value of ΔX_{HS} then is estimated to be 53 %, indicating a significant increase in the fraction of photoexcited HS molecules during the thermo-elastic

switching process at t = 37 ns. We acknowledge the large deviations in changes of the Bragg intensity observed between the +37 ns data (red lines) and the thermal equilibrium data (black lines) in Fig. 6e. However, we emphasize that our estimation, based on changes in peak intensity, aligns well with observations of radial position shifts and is consistent with previous literature[8,9,18,21]. This strongly supports the discussions and conclusions presented in our manuscript. On the other hand, from the experimentally observed lattice expansions at +100 ps (Fig. 5a), the known thermal expansion coefficients along the crystallographic axes, and heat deposited by pump laser, a temperature increase of about 40 K can be estimated (Supple. Note 5). This temperature elevation corresponds well with a HS fraction of ~ 50 %, matching with the HS fraction estimated by Bragg peak intensity changes.

Such large deviations in the structure factor primarily arise from differences between the out-of-equilibrium state at +37 ns and the thermal equilibrium state in thermally-induced SCO. The lattice volume expansion caused by photoheating results in a stressed crystal with an inhomogeneous distribution of unit cell volumes throughout the sample[21,22], as shown in Supple. Fig. 9 and Supple. Table 1. Supple. Fig. 9 and Supple. Table 1 illustrate the time evolution of the full width at half maximum (FWHM) of the 321 Bragg peak and the width changes observed between 313 and 353 K. No Bragg peak broadening was detected between 313 K and 353 K, since at 313 K and 353 K, the samples have good homogeneity, with most being in the LS state at 313 K or the HS state at 353 K. In contrast, a photoinduced HS fraction introduces a mixture of LS and HS states, leading to inhomogeneities in the sample. In recent publication[18], it monitored the thermal evolution of the 002 Bragg peak's width for the same sample. No broadening is observed after a complete thermally-induced SCO, matching our thermal measurements (Supple. Table 1). A notable increase in peak width is only observed near the phase transition temperature, where approximately half of the sample is in the LS state and the other half is in the HS state. This mixture of LS and HS states introduces inhomogeneity in the samples, resulting in peak broadening.

Therefore, the estimation of the excitation fraction from different Bragg peaks at +37 ns has a bigger distribution than estimation in the ultrafast timescale, which is before the lattice heating starts to dominate the changes in Bragg peak intensity and consistent with the estimation from the incident laser fluence. The inhomogeneous strain or disorder in the sample causes a loss of coherence in the scattered X-rays. This reduces constructive interference, leading to a decrease in peak intensity. Consequently, the excitation fraction estimation from Bragg peaks at +37 ns exhibits greater variability compared to the ultrafast timescale, where lattice heating has not yet dominated the changes in Bragg peak intensity.

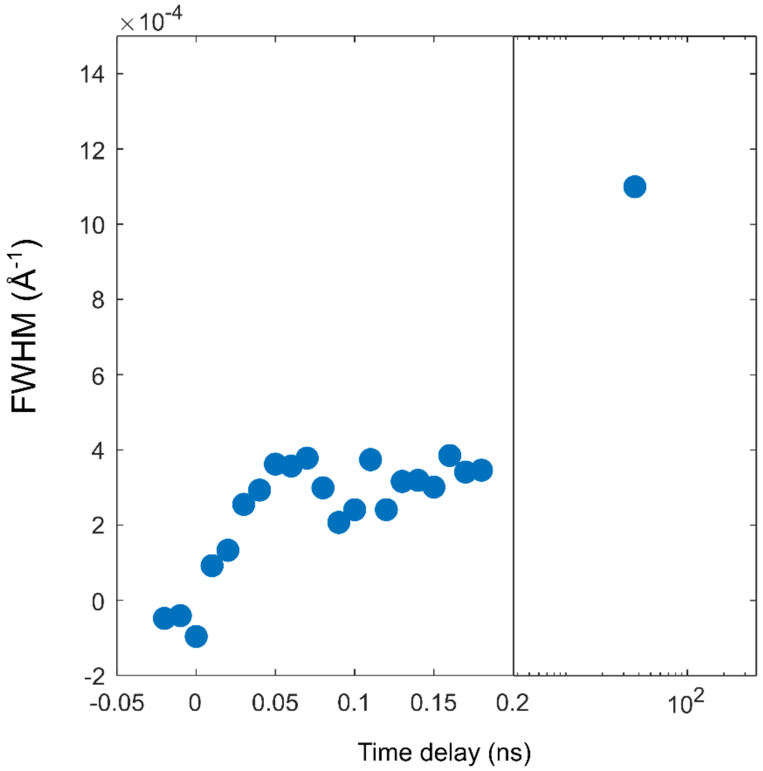

Supple. Fig. 9. Time evolution of the width of the 321 peak in FWHM. Source data are provided as a Source Data file.

Supple. Table 1. Changes of peak width of 321 Bragg peak at different time delays.

	Changes of peak width for 321 Bragg peak [$\times10^{-4}$ Å] ($\pm10^{-4}$ $Å^{-1}$)				
	+2 ps	+85 ps	+175 ps	+37 ns	Thermal SCO
FWHM	0	+3	+3	+11	0

Supple. Fig. 10 illustrates the HS fraction as a function of time based on the X-ray data. Regarding the molecule switching on the intermediate timescale, the excited molecules remain in the HS state, as the relaxation back to the low-spin (LS) state typically takes around 100 ns to 1 µs. This was demonstrated by previous optical works on the same compound[8] and other similar SCO system[9,21]. On the other hand, the same works[8,9,21] also present the unexcited molecules remain in the LS state until the second thermos-elastic SCO starts at around 1 ns as the existence of an energy barrier between the LS and HS states at the molecular scale in these previous studies.

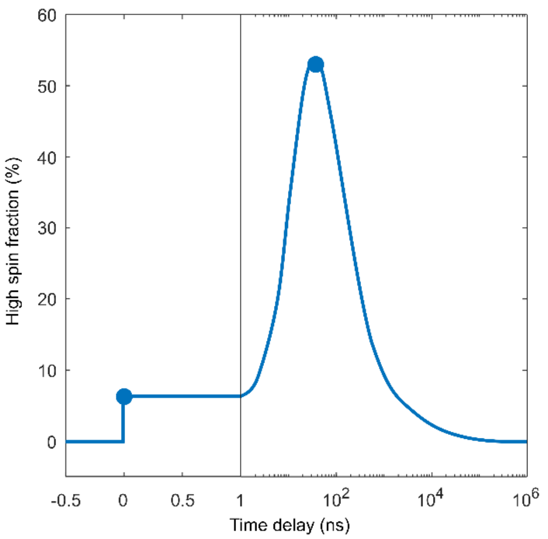

Supple. Fig. 10. **Time evolution of the fraction of photoswitched HS molecules.** Two fractions are based, determined from laser excitation conditions and changes in XRD Bragg peak intensities. The solid line, as a visual guide, is scaled based on previously measured optical and synchrotron XRD work[8,18]. Source data are provided as a Source Data file.

Supple. Discussion 1. Analysis of observed oscillations in the UED and XRD results

During our data analysis, we noticed weak oscillations in Fig. 2d of the UED results and Fig. 3b of XRD results. Similar oscillations have been observed in this ultrafast timescale for other SCO systems using optical spectroscopy[11] and X-ray absorption spectroscopy[10], where they were attributed to vibrational modes of the molecule, particularly the breathing and bending motions of the metal-ligand bonds. However, after careful analysis and a comprehensive comparison with existing literature, we conclude that these weak oscillations, given the noise level in our data, cannot offer sufficient experimental evidence to demonstrate significant physical relevance to the ultrafast dynamics investigated in this study. Further investigation of the potential oscillations on this or a similar system would require enhanced instrumental stability, increased statistical data and complementary ultrafast time-resolved techniques.

1. Oscillations observed in Fig. 2d of the UED results:
   a. The kinetic traces of Bragg peak intensities were fitted using a function that combines oscillation components with a biexponential decay, as shown in Supple. Fig. 11. Weak oscillations with a period of ~1 ps might be identified, but they are close to noise level. To study these oscillations with greater confidence, it requires better statistics from the instrument.
   b. Our findings indicate that the photoinduced SCO dynamics in our sample are similar to those observed in other Fe^{II} SCO systems. However, the oscillation period identified in our study does not match the 300 and 390 fs periods reported for similar SCO systems[10], which have been attributed to the breathing and bending modes of the Fe−N bonds in optical data.
   c. As shown in Supple. Fig. 12, we also conducted Fast-Fourier Transform (FFT) analysis on the changes in Bragg peak intensities from Fig. 2d after subtracting the fitted biexponential decay. In Supple. Fig. 12a, the residual changes in Bragg peak

intensities are displayed after removing the biexponential decay. The residual changes do not reveal any obvious oscillation, and some fluctuations are observed even before time zero. In Supple. Fig. 12b, the FFT results for individual Bragg peaks are shown. While some frequencies are detected, the results are heavily affected by noise, and no consistent frequency is observed across most or all peaks.

2. Oscillations observed in Fig. 3b of the XRD results: As shown in Supple. Fig. 13, a similar analysis was conducted on the changes in Bragg peak intensities from the XRD results. While some oscillations could be identified after fitting, they are weak and close to noise level.

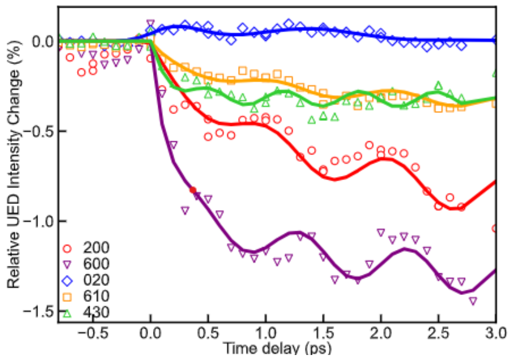

Supple. Fig. 11. **Kinetic traces of UED Bragg peak intensity for selected reflections from −1 to +4 ps.** The solid lines show the results of the fit to a bi-exponential decay functions with sinusoidal oscillations. Source data are provided as a Source Data file.

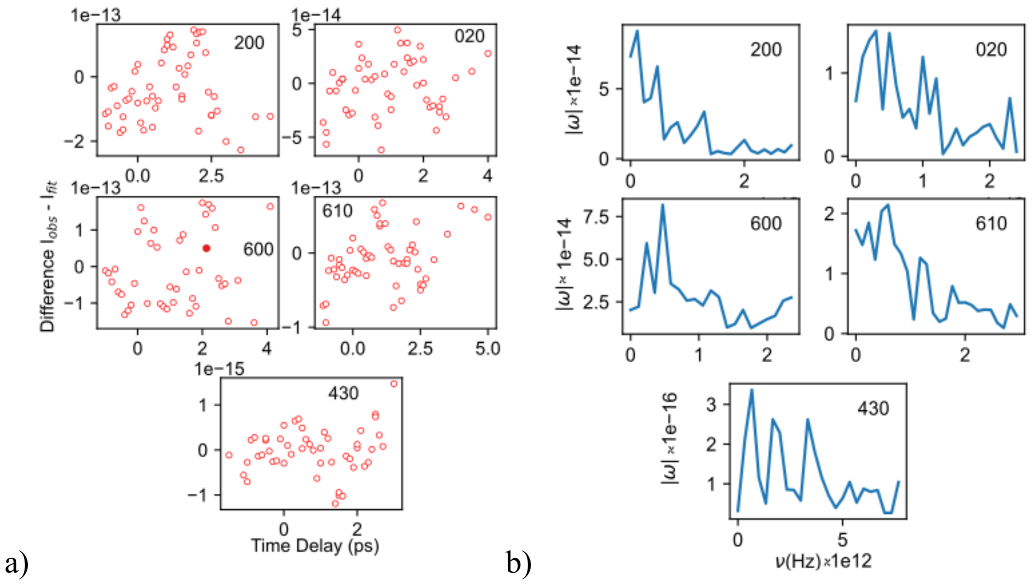

Supple. Fig. 12. **Fast-Fourier-Transform** a) Changes in Bragg peak intensities after removing the fitted exponential decay. b) Amplitude of the Fast-Fourier-Transform of the oscillations from different Bragg peaks. Source data are provided as a Source Data file.

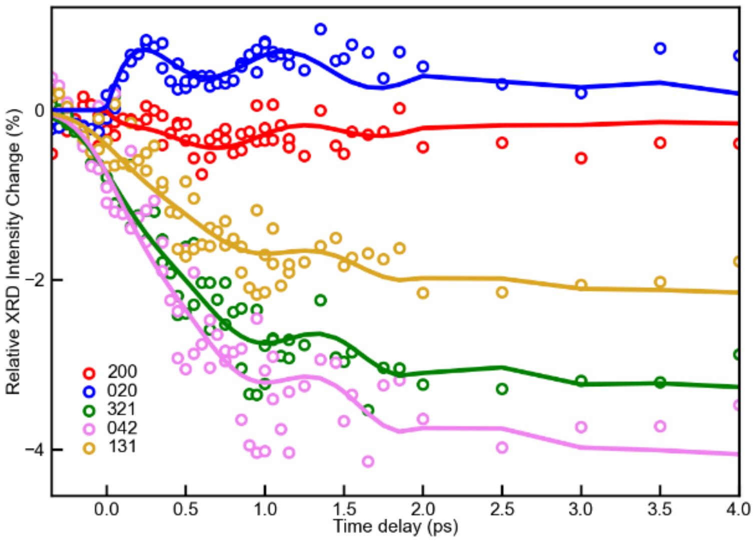

Supple. Fig. 13. **Kinetic traces of XRD Bragg peak intensity for selected reflections from −1 to +4 ps.** The solid lines show the results of the fit to a bi-exponential decay functions with sinusoidal oscillations. Source data are provided as a Source Data file.

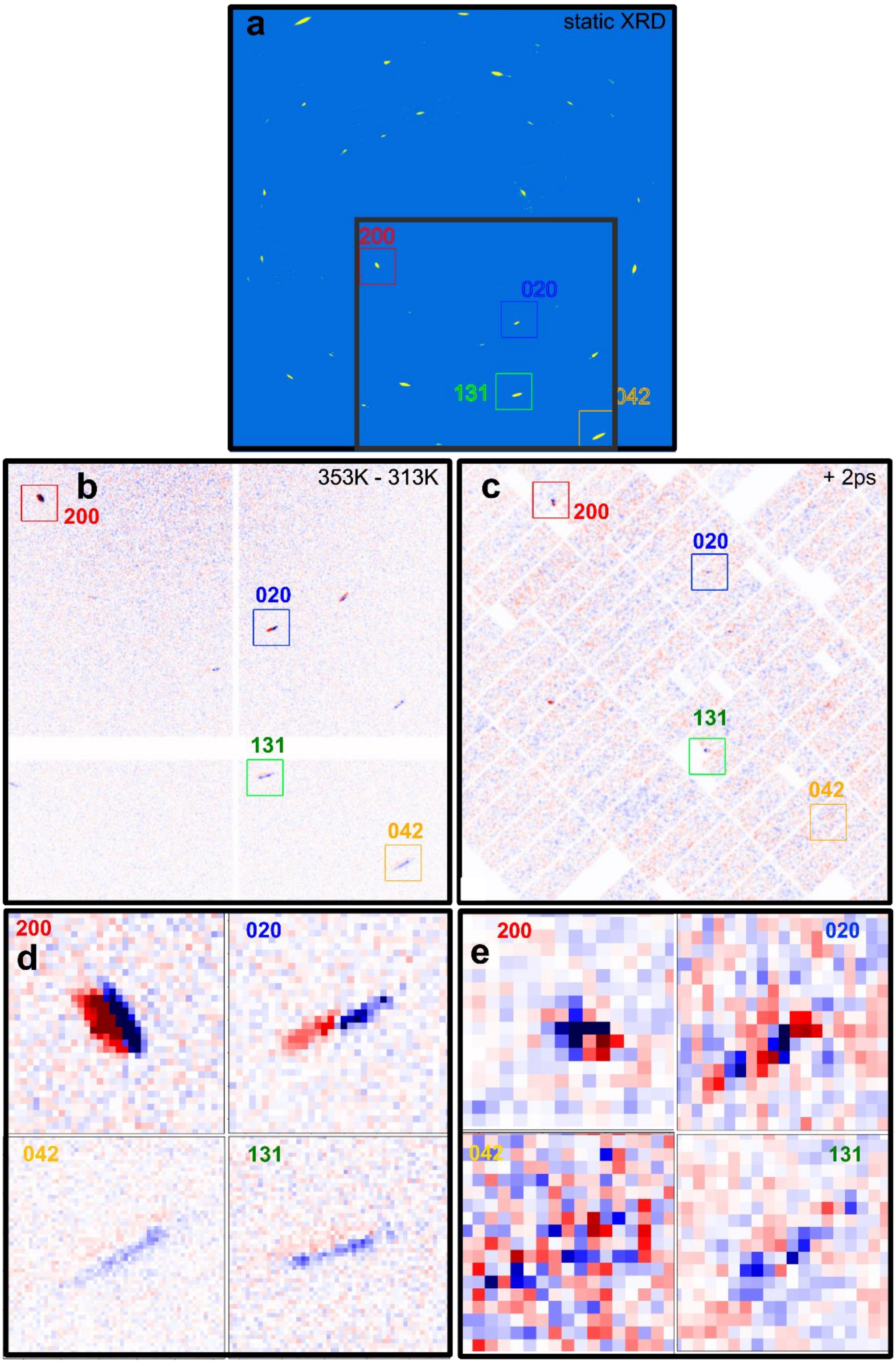

Supple. Fig. 14. Differences in thermally-induced SCO and photoinduced SCO at +2 ps. **a,** Static XRD diffraction pattern and color boxes show the positions of the selected Bragg peaks. **b,** difference between the diffraction patterns of the 313 K and 353 K**. c,** photoinduced signal measured after 2-ps time-delay. **b-c** are the selected area of the diffraction pattern indicated by the black rectangle in a. **d-e** show the selected area of the difference indicated by the color boxes in **a-c**.

Supplementary References:

1. Schmøkel, M. S., Kamiński, R., Benedict, J. B. & Coppens, P. Data scaling and temperature calibration in time-resolved photocrystallographic experiments. *Acta Crystallogr. A* **66**, 632–636 (2010).

2. Carbone, F., Yang, D.-S., Giannini, E. & Zewail, A. H. Direct role of structural dynamics in electron-lattice coupling of superconducting cuprates. *Proc. Natl. Acad. Sci. U. S. A.* **105**, 20161–20166 (2008).

3. Jiang, Y. *et al.* Direct observation of nuclear reorganization driven by ultrafast spin transitions. *Nat. Commun.* **11**, 1530 (2020).

4. Field, R., Liu, L. C., Gawelda, W., Lu, C. & Miller, R. J. D. Spectral Signatures of Ultrafast Spin Crossover in Single Crystal $[Fe^{II}(bpy)_3](PF_6)_2$. *Chem. - A Eur. J.* **22**, 5118–5122 (2016).

5. Jiang, Y. *et al.* Structural Dynamics upon Photoexcitation in a Spin Crossover Crystal Probed with Femtosecond Electron Diffraction. *Angew. Chem. Int. Ed.* **56**, 7130–7134 (2017).

6. Gao, M. *et al.* Mapping molecular motions leading to charge delocalization with ultrabright electrons. *Nature* **496**, 343–346 (2013).

7. Jiang, Y. *et al.* Direct observation of photoinduced sequential spin transition in a halogen-bonded hybrid system by complementary ultrafast optical and electron probes. *Nat. Commun.* **15**, 4604 (2024).

8. Ridier, K. *et al.* Finite Size Effects on the Switching Dynamics of Spin-Crossover Thin Films Photoexcited by a Femtosecond Laser Pulse. *Adv. Mater.* **31**, 1901361–1901361 (2019).

9. Volte, A. *et al.* Dynamical limits for the molecular switching in a photoexcited material revealed by X-ray diffraction. *Commun. Phys.* **5**, 168 (2022).

10. Lemke, H. T. *et al.* Coherent structural trapping through wave packet dispersion during photoinduced spin state switching. *Nat. Commun.* **8**, 15342 (2017).

11. Cammarata, M. *et al.* Sequential Activation of Molecular Breathing and Bending during Spin-Crossover Photoswitching Revealed by Femtosecond Optical and X-Ray Absorption Spectroscopy. *Phys. Rev. Lett.* **113**, 227402 (2014).

12. Gaffney, K. J. Capturing photochemical and photophysical transformations in iron complexes with ultrafast X-ray spectroscopy and scattering. *Chem. Sci.* **12**, 8010–8025 (2021).

13. Chergui, M. & Collet, E. Photoinduced Structural Dynamics of Molecular Systems Mapped by Time-Resolved X-ray Methods. *Chem. Rev.* **117**, 11025–11065 (2017).

14. Hauser, A. Light-Induced Spin Crossover and the High-Spin→Low-Spin Relaxation. in *Spin Crossover in Transition Metal Compounds II. Topics in Current Chemistry* Vol 234 (Springer, 2004).

15. Hauser, A., Enachescu, C., Daku, M. L., Vargas, A. & Amstutz, N. Low-temperature lifetimes of metastable high-spin states in spin-crossover and in low-spin iron(II) compounds: The rule and exceptions to the rule. *Coord. Chem. Rev.* **250**, 1642–1652 (2006).

16. Auböck., G. & Chergui, M. Sub-50-fs photoinduced spin crossover in $[Fe(bpy)_3]^{2+}$. *Nat. Chem.* **7**, 629–633 (2015).

17. Ridier, K. *et al.* Heat Capacity and Thermal Damping Properties of Spin-Crossover Molecules: A New Look at an Old Topic. *Adv. Mater.* **32**, 2000987 (2020).

18. Ridier, K. *et al.* Temporal Separation between Lattice Dynamics and Electronic Spin-State Switching in Spin-Crossover Thin Films Evidenced by Time-Resolved X-Ray Diffraction. *Adv. Funct. Mater.* 2403585 (2024).

19. Hauser, A. Intersystem crossing in the $[Fe(ptz)_6](BF_4)_2$ spin crossover system (ptz=1-propyltetrazole). *J. Chem. Phys.* **94**, 2741–2748 (1991).

20. Ridier, K., Nicolazzi, W., Salmon, L., Molnár, G. & Bousseksou, A. Sequential Activation of Molecular and Macroscopic Spin-State Switching within the Hysteretic Region Following Pulsed Light Excitation. *Adv. Mater.* **34**, 2105468 (2022).

21. Bertoni, R. *et al.* Elastically driven cooperative response of a molecular material impacted by a laser pulse. *Nat. Mater.* **15**, 606–610 (2016).

22. Collet, E. *et al.* Ultrafast spin-state photoswitching in a crystal and slower consecutive processes investigated by femtosecond optical spectroscopy and picosecond X-ray diffraction. *Phys. Chem. Chem. Phys.* **14**, 6192 (2012).
