# Supplementary material for: Capturing ultrafast molecular motions and lattice dynamics in spin crossover film using femtosecond diffraction methods
Source: Nat Commun. 2025 Feb 27;16:2043. doi: 10.1038/s41467-025-57202-0 (PMC11868369; doi:10.1038/s41467-025-57202-0)
Supplement: Supplementary file 2 — Description of Additional Supplementary Files [file 41467_2025_57202_MOESM2_ESM.pdf]

Description of Additional Supplementary Files:

Supplementary Data 1: Theoretical calculations revealed vibrational modes between 70 cm^{-1} and 200 cm^{-1}.

Supplementary Movie 1: Fe-ligand symmetric breathing mode

Supplementary Movie 2: Ligand bending mode

Supplementary Movie 3: Ligand torsion mode

Supplementary Movie 4: Out of phase Fe-ligand stretching
