# Supplementary figures and images for: Capturing ultrafast molecular motions and lattice dynamics in spin crossover film using femtosecond diffraction methods
Source: Nat Commun. 2025 Feb 27;16:2043. doi: 10.1038/s41467-025-57202-0 (PMC11868369; doi:10.1038/s41467-025-57202-0)

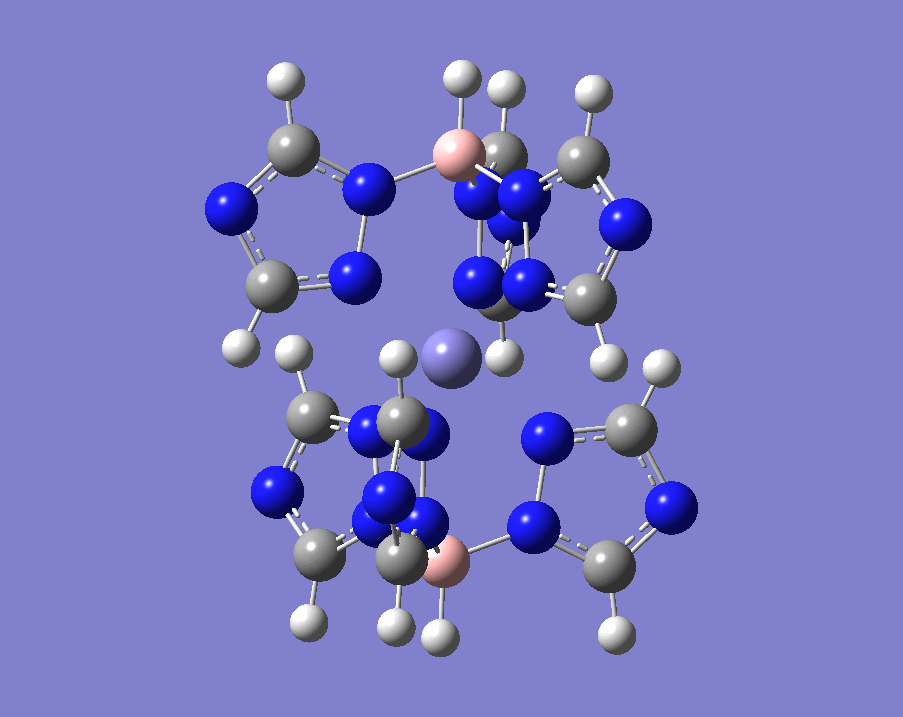

Supplement: Supplementary file 4 — Supplementary Movie 1 [file 41467_2025_57202_MOESM4_ESM.gif]

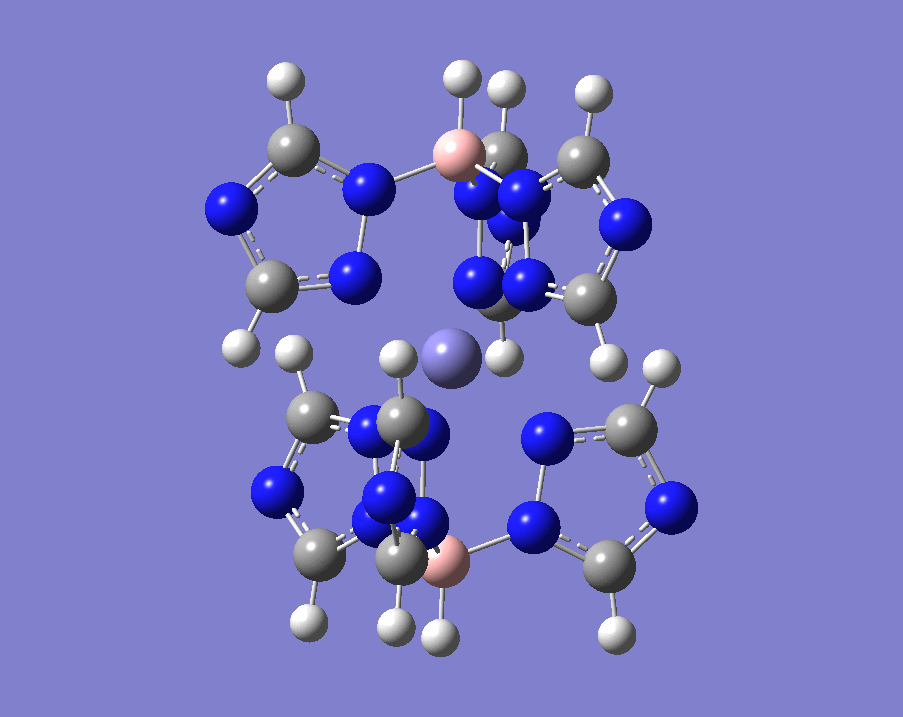

Supplement: Supplementary file 5 — Supplementary Movie 2 [file 41467_2025_57202_MOESM5_ESM.gif]

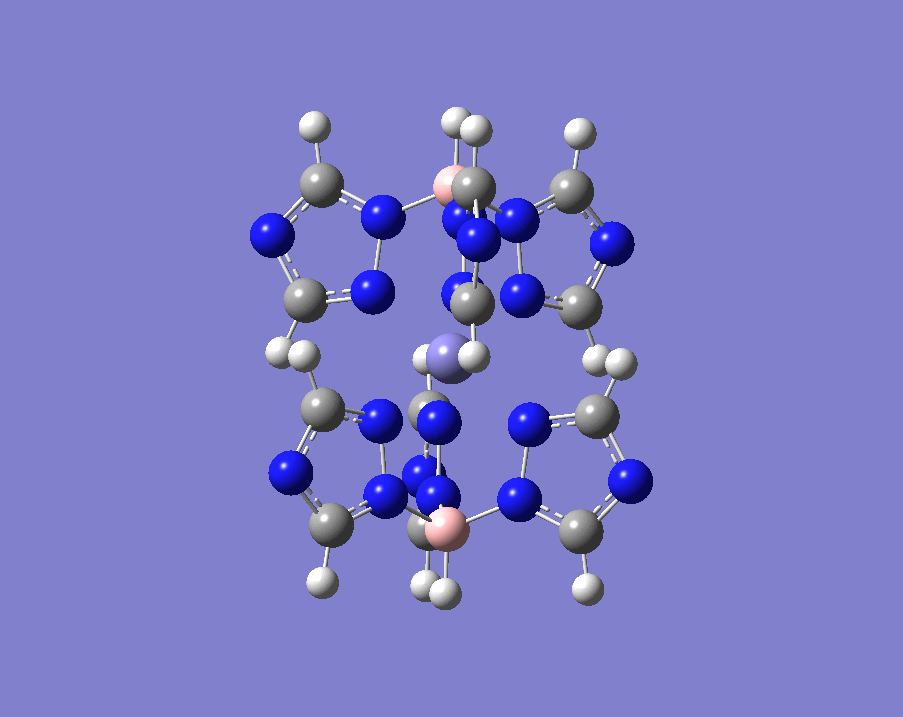

Supplement: Supplementary file 6 — Supplementary Movie 3 [file 41467_2025_57202_MOESM6_ESM.gif]

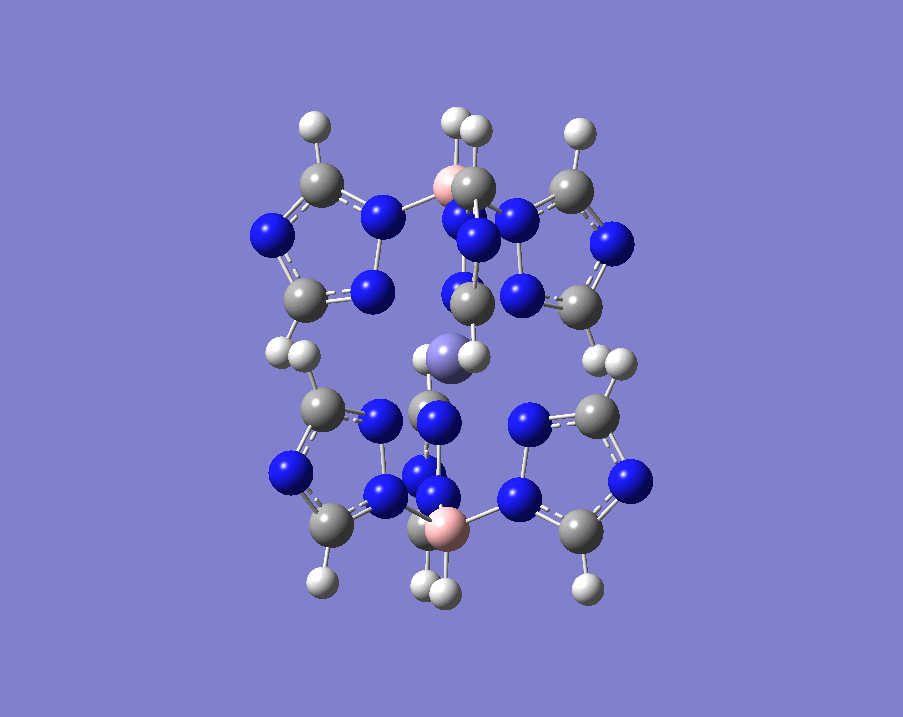

Supplement: Supplementary file 7 — Supplementary Movie 4 [file 41467_2025_57202_MOESM7_ESM.gif]
